# Glioblastoma cell differentiation trajectory predicts the immunotherapy response and overall survival of patients

**DOI:** 10.18632/aging.103695

**Published:** 2020-09-21

**Authors:** Zihao Wang, Xiaopeng Guo, Lu Gao, Yu Wang, Wenbin Ma, Bing Xing

**Affiliations:** 1 Department of Neurosurgery, Peking Union Medical College Hospital, Chinese Academy of Medical Sciences and Peking Union Medical College, Beijing, 100730, P. R. China

**Keywords:** glioblastoma, cancer cell differentiation, trajectory analysis, overall survival, immunotherapy response

## Abstract

Glioblastoma (GBM) is the most common and lethal primary brain tumor. In this study, we aimed to investigate the differentiation states of GBM cells and their clinical relevance. Integrated single-cell RNA-sequencing (scRNA-seq) data and bulk RNA-seq data from GBM samples were used for analysis. Two subsets of GBM cells in distinct differentiation states were characterized, and 498 GBM cell differentiation-related genes (GDRGs) were identified. GDRGs were significantly correlated with immune regulation and metabolic pathways. We classified the GBM patients into two groups based on the expression of GDRGs in tumors and found that the cell differentiation-based classification successfully predicted patient overall survival (OS), immune checkpoint expression and likelihood of immunotherapy response in GBMs. *FN1*, *APOE*, *RPL7A* and *GSTM2* were the 4 most significant survival-predicting GDRGs, and patients with different expression levels of each of these genes had distinct survival outcomes. Finally, a nomogram composed of the GDRG signature, age, pharmacotherapy, radiotherapy, IDH mutations and MGMT promoter methylation was generated and validated in two large GBM cohorts to predict GBM prognosis. This study highlights the significant roles of cell differentiation in predicting the clinical outcomes of GBM patients and their potential response to immunotherapy, suggesting promising therapeutic targets for GBM.

## INTRODUCTION

Glioblastoma (GBM) is the most common primary malignant brain tumor in adults, comprising 14.6% of all tumors and 48.3% of malignant tumors of the central nervous system (CNS) [1]. Due to the highly aggressive nature of GBM, neurological deficits, seizures and symptoms of intracranial hypertension occur rapidly in GBM patients within days or months [2]. Despite considerable advances in the development of treatments, including surgical resection, radiotherapy, and chemotherapy, little progress toward prolonged survival and better prognosis has been achieved over the last few decades [3]. The modest median overall survival (OS) time is approximately 14 months, and only 5% to 6.8% of GBM patients survive 5 years after diagnosis [1, 4]. Multiple clinical trials, including those on immunotherapy, have been conducted for GBM patients, but the results have been largely disappointing [5]. Prognosis predictors of GBM patients have been studied, and patient age, the extent of tumor resection, and several molecular alterations were reported as promising predictors [6–8]. Since autophagy plays a vital role in GBM development and progression, in our previous study, we generated a risk score nomogram based on 3 autophagy-related genes, *NRG1*, *ITGA3*, and *MAP1LC3A*, to predict the survival of GBM patients [9]. However, biomarkers and predictors for patient outcome and the immunotherapy response of GBMs have not been fully elucidated, and existing predictive models are far from satisfactory.

Multiple factors in the tumor microenvironment influence cancer cells during their differentiation from cancer stem cells (CSCs), leading to heterogeneous cell differentiation states and cell fates [10, 11]. Single-cell transcriptomics analysis has recently emerged as a powerful method to provide opportunities to characterize cell states and their transitions by simultaneously investigating the comprehensive nature of the genomes of an entire tumor sample at microscopic resolution [12]. Ordering such comprehensive tumor-constituting cells into trajectories helps us understand tumor cell subsets based on differentiation states and unveils the genetic cascades and related tumorigenic pathways accompanying cell fate specification [13]. Monocle 2, a recently generated algorithm that uses a reversed graph embedding strategy, is capable of accurately reconstructing single-cell trajectories according to the features of cell differentiation [14]. Therefore, by combining single-cell genomics and trajectory analyses, the cell subsets in differentiation states with distinct characteristics can be classified, and the new classifications have been reported to be correlated with diagnosis, progression and therapeutic outcomes in several diseases and tumors [15–19]. However, it remains unclear whether GBM cells are in different differentiation states and whether a new classification of GBM patients based on cell differentiation trajectories correlates with tumor biological behaviors and plays a role in predicting patient survival and the immunotherapy response.

Therefore, in this study, we included the transcriptomic data of human GBMs to verify our hypothesis that GBM cancer cells have diverse differentiation characteristics and that the classification of patients based on the differentiation features of GBM cells can predict the tumor immunotherapy response and patient survival. First, we identified two GBM cell subsets in distinct differentiation states by trajectory analysis using single-cell RNA-sequencing (scRNA-seq) data and identified significant GBM cell differentiation-related genes (GDRGs). Second, we explored the biological functions of the GDRGs and found that they are related to tumor immune regulation and metabolic pathways. Third, we included GBM patients from The Cancer Genome Atlas (TCGA) database and classified them based on the expression patterns of GDRGs and demonstrated that this GBM cell differentiation state-based classification method revealed significantly different OS outcomes and different likelihoods of an immunotherapy response. Then, *FN1*, *APOE*, *RPL7A* and *GSTM2* were identified as the 4 key OS-predicting GDRGs, and a clinically applicable prognostic nomogram using these 4 GDRGs and other clinicopathological variables was successfully developed for GBM patients. Finally, the above findings were validated using the GBM patient cohort from the Chinese Glioma Genome Atlas (CGGA) database. We identified distinct intratumoral GBM cell differentiation states and highlighted their essential role in predicting the clinical outcomes of GBM patients and tumor responses to immunotherapy.

## RESULTS

### Identification of 13 cell clusters in human GBMs using scRNA-seq data reveals high cell heterogeneity

**Figure 1:**
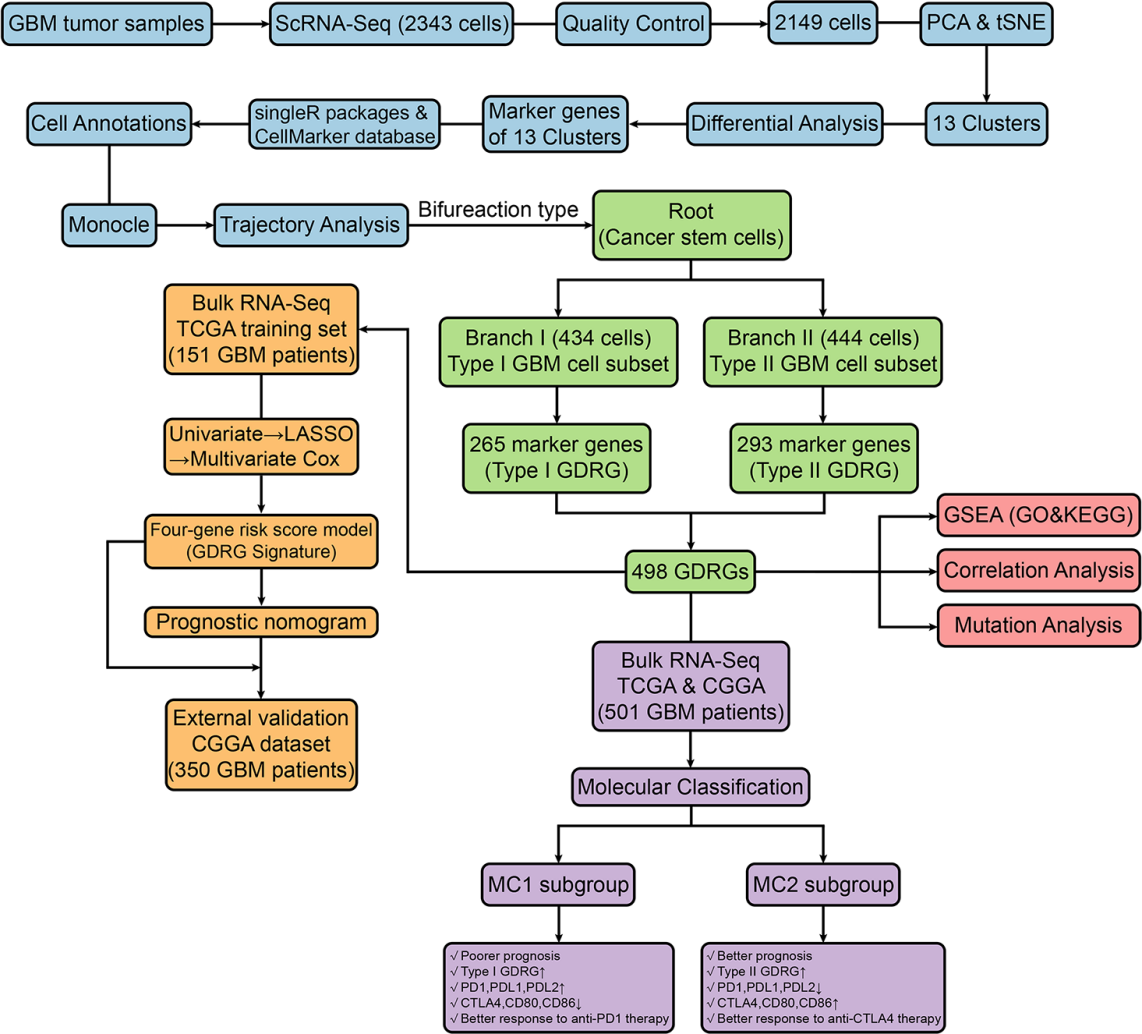
**Schematic diagram showing the study design and principal findings.**

**Figure 2:**
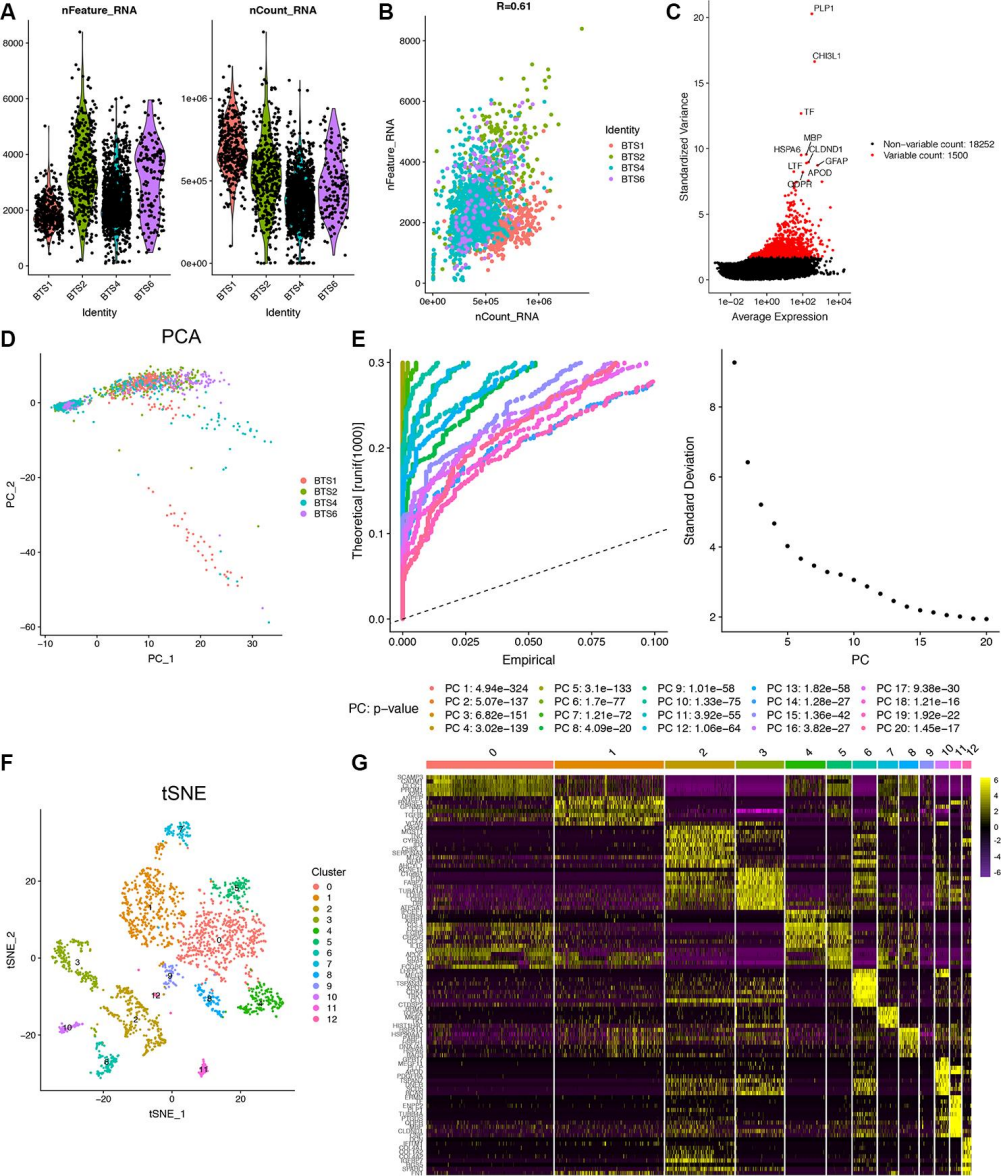
**Identification of 13 cell clusters with diverse annotations revealing high cellular heterogeneity in GBM tumors based on single-cell RNA-seq data.** (**A**) After quality control of the 2,343 cells from the tumor cores of 4 human GBM samples, 2,149 cells were included in the analysis. (**B**) The numbers of detected genes were significantly related to the sequencing depth, with a Pearson’s correlation coefficient of 0.61. (**C**) The variance diagram shows 19,752 corresponding genes throughout all cells from GBMs. The red dots represent highly variable genes, and the black dots represent nonvariable genes. The top 10 most variable genes are marked in the plot. (**D**) PCA did not demonstrate clear separations of cells in GBMs. (**E**) PCA identified the 20 PCs with an estimated P value < 0.05. (**F**) The tSNE algorithm was applied for dimensionality reduction with the 20 PCs, and 13 cell clusters were successfully classified. (**G**) The differential analysis identified 8,025 marker genes. The top 20 marker genes of each cell cluster are displayed in the heatmap. A total of 96 genes are listed beside of the heatmap after omitting the same top marker genes among clusters. The colors from purple to yellow indicate the gene expression levels from low to high.

A schematic diagram of the study design and principal findings is shown in Figure 1. Following the quality control standard and the normalization of GBM scRNA-seq data, 194 low-quality cells were excluded, and 2,149 cells from GBM cores were included in the analysis (Figure 2A). The number of genes detected was significantly related to the sequencing depth (Figure 2B). A total of 19,752 corresponding genes were included, and the variance analysis revealed 1,500 highly variable genes (Figure 2C). Principal component analysis (PCA) was performed to identify available dimensions and screen correlated genes. The top 20 significantly correlated genes are displayed as dot plots and heatmaps in Supplementary Figure 1. However, the PCA results did not demonstrate clear separations among cells in human GBMs (Figure 2D). We selected 20 principal components (PCs) with an estimated P value < 0.05 for subsequent analysis (Figure 2E).

**Figure 3:**
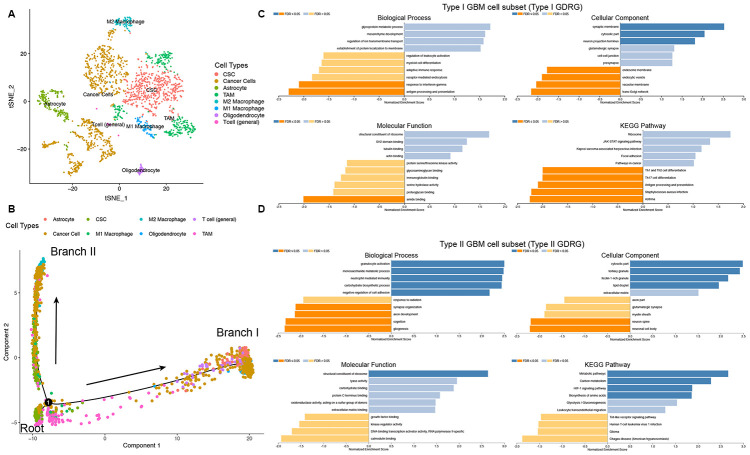
**Cell annotation, trajectory analysis and GSEA of two GBM cell subsets with distinct differentiation patterns.** (**A**) All 13 clusters of cells in GBMs were annotated by singleR and CellMarker according to the composition of the marker genes. (**B**) Trajectory analysis revealed two subsets of GBM cells with distinct differentiation patterns. GBM CSCs were mainly located in the root, whereas GBM cells were located in either branch. Branch I GBM cells were defined by the type I GBM cell subset (434 GBM cells). Branch II GBM cells were defined by the type II GBM cell subset (444 GBM cells). (**C** and **D**) GSEA of type I and II GBM cell subsets was performed to identify related molecular mechanisms and pathways. An FDR ≤ 0.05 was considered statistically significant.

Afterwards, the t-distributed stochastic neighbor embedding (tSNE) algorithm was applied, and cells in human GBMs were successfully classified into 13 separate clusters (Figure 2F). Differential expression analysis was performed, and a total of 8,025 marker genes from all 13 clusters were identified (Figure 2G). According to the expression patterns of the marker genes, these clusters were annotated by singleR and CellMarker (Figure 3A). Cluster 0, containing 518 cells, was annotated as GBM CSCs; clusters 1, 2, 6 and 10, containing 878 cells, were annotated as GBM cancer cells or GBM cells; cluster 3, containing 196 cells, was annotated as astrocytes; cluster 11, containing 44 cells, was annotated as oligodendrocytes; clusters 4, 5 and 9, containing 319 cells, were annotated as tumor-associated macrophages; cluster 8, containing 77 cells, was annotated as typical M1 macrophages; cluster 7, containing 81 cells, was annotated as typical M2 macrophages; and cluster 12, containing 36 cells, was annotated as T cells.

### GBM cells can be divided into two subsets with distinct differentiation patterns, with their GDRGs correlating with immune and metabolic pathways

Trajectory analysis was performed to project all cells from GBMs onto one root and two branches, termed branches I and II (Figure 3B). The results showed that GBM CSCs were mainly located in the root, whereas GBM cells were mostly located in the branches. Interestingly, GBM cells in branch I, defined here as type I GBM cells, were all from clusters 2, 6, and 10 (434 cells), and GBM cancer cells in branch II, defined here as type II GBM cells, were all from cluster 1 (444 cells). The degree of differentiation of cells in the type I and type II GBM subsets varied significantly. The branch-dependent marker genes of type I and type II GBM cells were determined to be GDRGs. Differential analysis was performed, and 265 marker genes were identified as type I GDRGs, and 193 marker genes were identified as type II GDRGs. Therefore, a total of 498 GDRGs were ultimately identified in GBM (Supplementary Table 1).

Gene set enrichment analysis (GSEA) was performed to identify related molecular mechanisms and pathways of GBM cells with distinct differentiation patterns. The results revealed that type I GDRGs were significantly negatively correlated with the regulation of immune processes, such as antigen processing and immune cell differentiation (Figure 3C), and that type II GDRGs were significantly positively correlated with metabolism-related pathways, such as carbon metabolism, amino acid biosynthesis, glycolysis, and gluconeogenesis (Figure 3D). These findings from GDRGs indicate that GBM cells in distinct differentiation states demonstrate distinct tumor biology characteristics, which might provide new evidence for the molecular signatures of GBMs, including both intrinsic properties and the regulation of related pathways.

**Figure 4:**
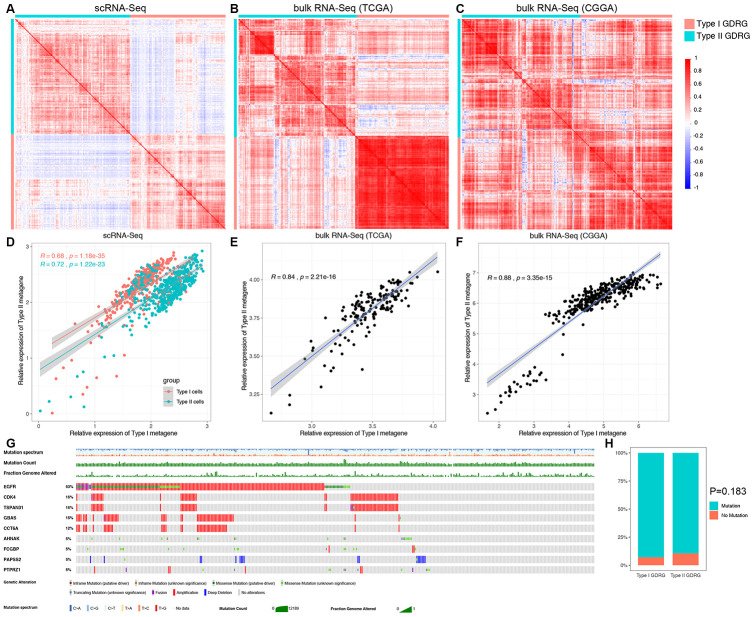
**Correlation analysis and somatic mutation analysis of the two subtypes of GDRGs.** The correlation heatmaps, which were generated to determine whether the observed GBM cell subsets could be identified using bulk RNA-seq data, demonstrated that the type I GDRGs were highly correlated in both scRNA-seq data (**A**) and bulk RNA-seq data, including the TCGA (**B**) and CGGA cohorts (**C**). The same result was observed for the type II GDRGs (**A**–**C**). The correlation analysis demonstrated that the expression of type I and type II metagenes was significantly correlated in both scRNA-seq data (**D**) and bulk RNA-seq data, including the TCGA (**E**) and CGGA cohorts (**F**). (**G**) OncoPlot analysis of the somatic mutation status of the GDRGs in the TCGA cohort revealed the top 9 mutated genes with mutation frequencies ≥ 5%. (**H**) Mutation frequencies of type I and type II GDRGs. A total of 246 genes (92.8%) were mutated in type I GDRGs, and 262 genes (89.4%) were mutated in type II GDRGs (P=0.183).

Then, we determined whether the observed GBM cell subsets could be identified using bulk RNA-seq data. As shown in Figure 4A–4C, the correlation analyses demonstrated that the type I GDRGs were highly correlated between scRNA-seq and bulk RNA-seq data from both the TCGA and CGGA databases, as were the type II GDRGs. These findings suggest that type I and type II GBM cells can also be identified by GDRG expression using bulk RNA-seq data, since these highly correlated genes could indicate a common cellular origin.

### GDRGs in the two GBM cell subsets are functionally correlated and mostly mutated

To determine whether the gene profiles originating from different GBM cell subsets were functionally correlated, we utilized metagenes to represent the overall expression patterns of the corresponding gene profile. The expression of type I and type II metagenes, consisting of type I and type II GDRGs, respectively, were derived from the weighted averages of the expression of the constituent genes. As shown in Figure 4D–4F, the results demonstrated significantly strong correlations between type I and type II metagene expression in both scRNA-seq and bulk RNA-seq data, indicating that type I and type II GDRGs are functionally correlated even though they originate from distinct GBM subsets with different differentiation patterns.

We also analyzed the somatic mutation statuses of the GDRGs in the TCGA cohort. Most GDRGs (90.8%, 452/498) harbored mutations, and the top 9 mutated GDRGs with mutation frequencies ≥ 5% in GBM patients are shown in Figure 4G. Epidermal growth factor receptor *(EGFR)* exhibited the highest mutation frequency (53%), followed by *CDK4* (16%) and *TSPAN31* (16%). There were 246 genes (92.8%) exhibiting mutations among type I GDRGs and 262 genes (89.4%) exhibiting mutations among type II GDRGs. The mutation frequencies were not significantly different between the two groups (P=0.183, Figure 4H). However, 8 of the top 9 mutated GDRGs were type II GDRGs, and only 1 was a type I GDRG. These findings demonstrate the high mutation status heterogeneity in GDRGs, suggesting the pivotal roles of GDRGs in the development and progression of GBMs.

### GDRG-based classification of GBM patients correlates with different OS outcomes and clinicopathological characteristics

**Figure 5:**
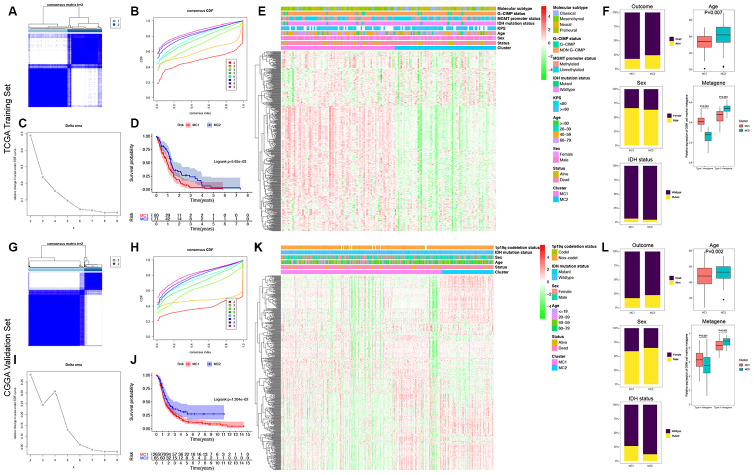
**Identification and validation of the GDRG-based classification of GBM patients.** Consensus clustering matrix for k = 2, which was the optimal cluster number in the TCGA training cohort (**A**) and CGGA validation cohort (**G**). (**B** and **H**) CDF curves of the consensus score (k = 2-9) in the TCGA and CGGA cohorts. (**C** and **I**) Relative change in the area under the CDF curve (k = 2-9) in the TCGA and CGGA cohorts. Kaplan-Meier survival analyses of the patients with MC1 and MC2 GBMs in the TCGA (**D**) and CGGA (**J**) cohorts, indicating that the patients with MC1 GBMs had poorer OS than those with MC2 GBMs. Heatmap and clinicopathological features of the two MCs in the TCGA (**E**) and CGGA (**K**) cohorts showing that the expression levels of the type I GDRG metagene were significantly higher and the levels of the type II GDRG metagene were significantly lower in patients with MC1 GBMs than in patients with MC2 GBMs. (**F** and **L**) Comparisons of the clinicopathological variables and type I and II metagenes between the two MCs of GBM patients in the TCGA and CGGA cohorts.

To establish a classification of GBMs based on the expression patterns of GDRGs, machine learning-based unsupervised consensus clustering was performed on 151 GBM patients from the TCGA database. According to the relative change in the area under the cumulative distribution function (CDF) curve and consensus heatmap, the optimal number of clusters was determined to be two (k value = 2), and no appreciable increase was observed in the area under the CDF curve (Figure 5A–5C). Thus, all GBMs were classified into two groups: 80 (53.0%) in molecular cluster 1 (MC1) and 71 (47.0%) in molecular cluster 2 (MC2). Kaplan-Meier survival analysis indicated that patients with MC1 GBMs experienced significantly poorer OS than patients with MC2 GBMs (log-rank P=5.65×10^-3^; Figure 5D).

Afterwards, we validated the GDRG-based classification in the CGGA cohort. As shown in Figure 5G–5I, the optimal number of clusters was also determined to be two (k value = 2), and the patients were also classified as MC1 (265 patients, 75.7%) and MC2 (85 patients, 24.3%). Kaplan-Meier survival analysis also demonstrated that patients with MC1 GBMs had poorer OS than patients with MC2 GBMs (log-rank P=1.26×10^-2^; Figure 5J).

Additionally, we compared the expression patterns of the GDRGs and clinicopathological characteristics between two MCs of patients in the TCGA cohort. As shown in Figure 5E and 5F, the expression levels of the type I GDRG metagene were significantly higher (P<0.001) and the expression levels of the type II GDRG metagene were significantly lower (P<0.001) in patients with MC1 GBMs than in patients with MC2 GBMs. The same findings were also observed in the CGGA validation cohort (Figure 5K and 5L). Hence, we hypothesize that MC1 patients correspond mostly to the functional properties of the type I GBM subset and MC2 patients correspond mostly to the functional properties of the type II GBM subset.

The demographics and clinicopathological characteristics of patients with MC1 and MC2 GBMs are shown in Table 1. Compared with MC2 patients, MC1 patients were significantly younger in both the TCGA cohort (P=0.007) and CGGA cohort (P=0.002). However, no significant differences were observed in other variables between the two MCs of patients (all P > 0.05). Overall, the above findings indicate that this GDRG-based classification of GBM patients is robust and reliable across different populations, and different survival outcomes can be clearly discriminated according to this classification.

**Table 1:** Demographics and clinicopathological characteristics of GBM patients in the TCGA training cohort and CGGA validation cohort in the MC1 and MC2 subgroups based on the molecular classification.

**Variable**	**TCGA training cohort**			**CGGA validation cohort**		
	**Total (n=151)**	**MC1 (n=80)**	**MC2 (n=71)**	**Total (n=350)**	**MC1 (n=265)**	**MC2 (n=85)**
Age (years)	59.6±13.7	58.7±13.3	60.6±14.1	48.1±13.3	47.0±13.8	51.5±11.1
Sex						
Female	53	27	26	139	109	30
Male	98	53	45	211	156	55
KPS						
< 80	32	16	16	NA		
≥ 80	81	40	41	NA		
NA	38	24	14	NA		
Pharmacotherapy					
TMZ	64	33	31	61 (No)	51	10
TMZ+BEV	26	17	9	269 (Yes)	201	68
Others (No TMZ)	19	11	8	-	-	-
No or NA	42	19	23	20 (NA)	13	7
Radiotherapy						
No	22	15	7	48	39	9
Yes	122	64	58	283	214	69
NA	7	1	6	19	12	7
Surgery						
Biopsy only	16	6	10	NA		
Tumor resection	135	74	61	NA		
IDH mutation status						
Wild-type	147	75	68	270	195	75
Mutant	8	5	3	80	70	10
MGMT promoter status					
Methylated	66	40	26	NA		
Unmethylated	85	40	45	NA		
TERT status						
Wild-type	146	78	68	NA		
Mutant	5	2	3	NA		
BRAF status						
Wild-type	146	76	70	NA		
Mutant	5	4	1	NA		
ATRX status						
Wild-type	140	72	68	NA		
Mutant	11	8	3	NA		
EGFR status						
Wild-type	97	56	41	NA		
Mutant	54	24	30	NA		
G-CIMP status						
Non G-CIMP	140	74	66	NA		
G-CIMP	11	6	5	NA		
Molecular subtype						
Classical	38	11	27	NA		
Neural	26	15	11	NA		
Mesenchymal	49	40	9	NA		
Proneural	38	14	24	NA		
1p/19q status						
Noncodeletion	NA			323	240	83
Codeletion	NA			17	15	2
NA	NA			10	10	0

“Others (No TMZ)” in pharmacotherapy included PCV, PCV+BEV, and other drugs, including avastin, carmustine, and irinotecan.

MC, molecular cluster; GBM, glioblastoma; NA, not available; KPS, Karnofsky performance status; TMZ, temozolomide; BEV, bevacizumab; PCV, procarbazine lomustine vincristine.

### GDRG-based classification of GBM patients correlates with the expression patterns of immune checkpoints and different likelihoods of an immunotherapy response

**Figure 6:**
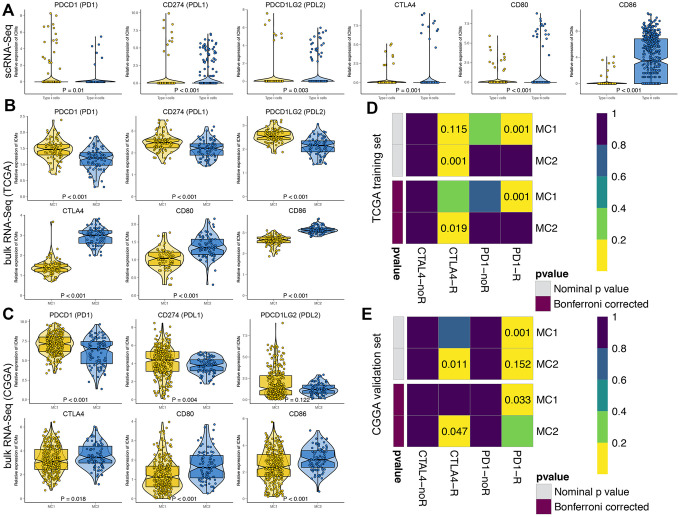
**Predictions of the immunotherapy response in GBM patients.** The violin plots present the expression of 6 principal immune checkpoint molecules, namely, PDCD1 (PD1), CD274 (PDL1), PDCD1LG2 (PDL2), CTLA4, CD80, and CD86, in scRNA-seq data (**A**) and bulk RNA-seq data, including the TCGA (**B**) and CGGA cohorts (**C**). Subclass mapping analysis was used to predict the likelihood of the clinical response to anti-PD1 and anti-CTLA4 therapy for MC1 and MC2 GBM patients from the TCGA (**D**) and CGGA (**E**) cohorts. R represents immunotherapy responders, while noR represents immunotherapy nonresponders. A Bonferroni-corrected P value < 0.05 was considered statistically significant.

The expression of 6 main immune checkpoints, namely, PDCD1 (PD1), CD274 (PDL1), PDCD1LG2 (PDL2), CTLA4, CD80 and CD86, was determined and compared between two GBM cell subsets and two MCs of GBM patients (Figure 6). In terms of scRNA-seq data, PD1, PDL1 and PDL2 were highly expressed in type I GBM cells, whereas CTLA4, CD80 and CD86 were highly expressed in type II GBM cells (Figure 6A). In terms of bulk RNA-seq data, PD1, PDL1 and PDL2 were highly expressed in MC1 GBM patients whereas CTLA4, CD80 and CD86 were highly expressed in MC2 GBM patients in both the TCGA database (Figure 6B) and CGGA database (Figure 6C).

Afterwards, the tumor immune dysfunction and exclusion (TIDE) algorithm was used to predict the likelihood of an immunotherapy response. According to the results from the TCGA training cohort, MC2 GBM patients (43.7%, 31/71) were more likely to respond to immunotherapy than MC1 patients (20.0%, 16/80, P = 0.003). Similarly, in the CGGA validation cohort, MC2 GBM patients (45.9%, 39/85) were more likely to respond to immunotherapy than MC1 patients (37.6%, 62/165, P < 0.001).

Then, SubMap analysis was performed to predict the likelihood of a clinical response to PD1 and CTLA4 inhibitors in the two MCs of GBM patients. MC1 GBM patients in both the TCGA and CGGA cohorts were more sensitive to anti-PD1 therapies, with Bonferroni-corrected P values of 0.001 and 0.033, respectively, while MC2 GBM patients were more sensitive to anti-CTLA4 therapies, with Bonferroni-corrected P values of 0.019 and 0.047, respectively (Figure 6D and 6E).

### FN1, APOE, RPL7A and GSTM2 are the 4 most significant survival-predicting GDRGs in human GBMs

Univariate Cox analysis was performed, and 45 prognosis-associated GDRGs were identified in the TCGA training set. Least absolute shrinkage and selection operator (LASSO) followed by multivariate Cox analysis was then performed, and 4 significant survival-predicting GDRGs were identified (Supplementary Figure 2): fibronectin 1 (*FN1*, HR=5.28, P<0.001), apolipoprotein E (*APOE*, HR=0.39, P=0.001), ribosomal protein L7a (*RPL7A*, HR=0.27, P=0.021), and glutathione S-transferase mu 2 (*GSTM2*, HR=0.41, P=0.042). The expression levels of the 4 most significant prognostic GDRGs in both the scRNA-seq and bulk RNA-seq profiles are shown in Supplementary Figure 3. *FN1* was significantly upregulated in GBM cells and T cells (general); *APOE* was significantly upregulated in GBM cells, CSCs and macrophages; *RPL7A* was significantly upregulated in GBM cells, CSCs and macrophages; and *GSTM2* was significantly upregulated in GBM cells and astrocytes (Supplementary Figure 3A). Furthermore, the expression of these 4 GDRGs was validated using the Gene Expression Profiling Interactive Analysis (GEPIA) database, which includes 163 GBM (TCGA) samples and 207 normal (GTEx) samples. We found that all 4 survival-predicting GDRGs were upregulated in GBMs compared to normal tissues (Supplementary Figure 3B).

Kaplan-Meier survival analysis demonstrated that high expression of *FN1* and low expression of *APOE*, *GSTM2* and *RPL7A* were associated with poor OS in GBM patients (Supplementary Figure 3C).

### Generation and validation of the GDRG-based prognostic risk score model to predict GBM patient survival

**Figure 7:**
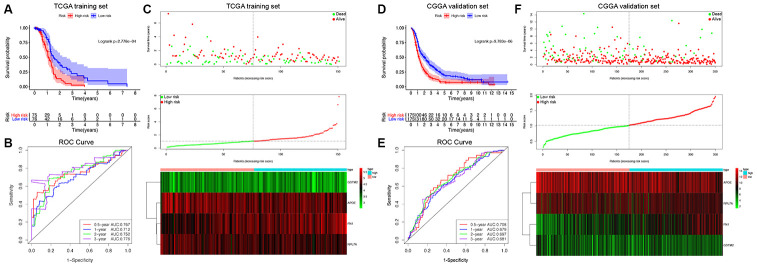
**Survival analysis, prognostic performance and risk score analysis of the GDRG-based risk score model in GBM patients.** Kaplan-Meier survival analysis was performed to estimate the OS of high-risk and low-risk patients in the TCGA training cohort (**A**) and CGGA validation cohort (**D**). The high-risk groups had significantly poorer OS than the low-risk groups. Time-dependent ROC curve analysis was performed to evaluate the prognostic performance of the GDRG signature for predicting the 0.5-, 1-, 2-, and 3-year OS rates in the TCGA (**B**) and CGGA cohorts (**E**). Risk score analysis of the GDRG signatures in the TCGA (**C**) and CGGA (**F**) cohorts were calculated, and the patients were divided into either a high-risk group or a low-risk group using the median value of the risk score as the cutoff value. Upper panel: Patient survival status and time distributed by the risk score. Middle panel: Risk score curves of the GDRG signatures. Bottom panel: Heatmaps of the expression levels of the 4 GDRGs in the GBM samples. The colors from green to red indicate the gene expression levels from low to high.

The prognostic risk score model was developed based on the above 4 GDRGs with the following formula: Risk score = Exp_*FN1*_ × 1.66 + Exp_*APOE*_ × (-0.93) + Exp_*RPL7A*_ × (-1.30) + Exp_*GSTM2*_ × (-0.90). Risk scores were calculated for all patients in the TCGA training set, and the patients were divided into either a high-risk (high score) group or a low-risk (low score) group using the median value of the risk score as the cutoff value (Figure 7C). Kaplan-Meier survival analysis demonstrated that patients in the high-risk group had significantly poorer OS than those in the low-risk group (log-rank, P = 2.778×10^-4^; Figure 7A). The C-index of the GDRG signature for OS prediction was 0.781 (95% CI=0.742 to 0.820). Time-dependent receiver operating characteristic (ROC) analysis also indicated that the GDRG signature showed excellent performance in predicting the 0.5-, 1-, 2- and 3-year OS rates, with respective area under the curve (AUC) values of 0.767, 0.712, 0.752 and 0.776 (Figure 7B).

Then, the predictive formula was validated using the CGGA cohort in a similar manner. As shown in Figure 7F, all GBM patients were classified into high-risk or low-risk groups. Consistent with the results from the TCGA training set, the survival analysis also demonstrated that patients in the high-risk group had significantly poorer OS than patients in the low-risk group (log-rank, P = 9,783×10^-6^; Figure 7D). The C-index of the GDRG signature was 0.715 (95% CI=0.676 to 0.754). Time-dependent ROC analysis also suggested favorable values in predicting OS in the CGGA validation set (Figure 7E). These results indicate that the GDRG-based prognostic risk score model can serve as a reliable prognostic predictor for different populations of GBM patients.

### Development and validation of the clinically applicable prognostic nomogram with the GDRG signature and clinicopathological parameters

**Figure 8:**
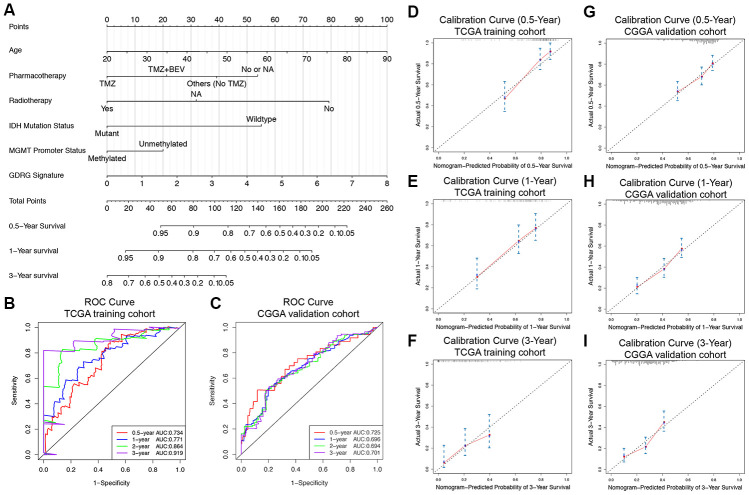
**Prognostic nomogram to predict the 0.5-, 1-, and 3-year OS of GBM patients.** (**A**) Nomogram model to predict the prognosis of GBM patients based on the TCGA training cohort. Age, pharmacotherapy, radiotherapy, IDH mutation status, MGMT promoter methylation status, and the GDRG signature were included in the prediction model. The prognostic performance of the nomogram demonstrated by the time-dependent ROC curve for predicting the 0.5-, 1-, and 3-year OS rates in the TCGA training cohort (**B**) and CGGA validation cohort (**C**). Calibration plots of the prognostic nomogram for predicting OS at 0.5, 1, and 3 years in the TCGA (**D**–**F**) and CGGA (**G**–**I**) cohorts. Actual survival is plotted on the y-axis, and nomogram-predicted probability is plotted on the x-axis.

To investigate whether the prognostic significance of the GDRG signature is independent of the other clinicopathological variables in predicting the survival of GBM patients, univariate and multivariate Cox regression analyses were performed, and the results demonstrated that the GDRG signature was independently associated with OS in both the TCGA and CGGA cohorts (Table 2). Finally, a prognostic nomogram was successfully developed to provide a clinically applicable quantitative approach for individual OS prediction. Age, pharmacotherapy, radiotherapy, IDH mutation status, MGMT promoter methylation status, and the GDRG signature were included in the final OS prediction model (Figure 8A). The C-index of the prognostic nomogram was 0.896 (95% CI=0.857 to 0.935). Time-dependent ROC analysis revealed excellent predictive abilities for the 0.5-, 1-, 2- and 3-year OS rates, with AUC values of 0.734, 0.771, 0.864 and 0.919, respectively (Figure 8B). The calibration plots showed excellent agreement between the predicted 0.5-, 1- and 3-year OS rates and the actual observations in the TCGA cohort (Figure 8D–8F). Then, the prognostic model was validated in the CGGA cohort, with a C-index of 0.729 (95% CI=0.690 to 0.768). The time-dependent AUCs for the 0.5-, 1-, 2-, and 3-year OS rates with the prognostic nomogram were 0.725, 0.696, 0.694, and 0.701, respectively (Figure 8C). The calibration plots also showed excellent agreement between the OS predictions and the actual observations for the probabilities of the 0.5-, 1- and 3-year survival rates in the validation set (Figure 8G–8I). The above findings suggest the appreciable reliability of the prognostic nomogram for OS prediction that can be applied in different populations of GBM patients.

**Table 2:** Univariate and multivariate Cox proportional hazards analyses of clinicopathological variables and GDRG signatures in the TCGA GBM training cohort and CGGA GBM validation cohort.

**Variable**	**TCGA training cohort (n=151)**				**CGGA validation cohort (n=350)**			
	**Univariate analysis**		**Multivariate analysis**		**Univariate analysis**		**Multivariate analysis**	
	**HR (95% CI)**	**P value**	**HR (95% CI)**	**P value**	**HR (95% CI)**	**P value**	**HR (95% CI)**	**P value**
**Age**	1.028 (1.013-1.044)	**1.98e-04**	1.017 (1.000-1.035)	**4.59e-02**	1.078 (1.048-1.108)	**8.35e-05**	1.006 (1.001-1.011)	**1.90e-02**
**Sex (Female/Male)**	0.916 (0.626-1.341)	0.65	-	-	1.063 (0.837-1.350)	0.61	-	-
**KPS (<80/≥80/NA)**	0.926 (0.696-1.233)	0.59	-	-	NA		NA	
**Pharmacotherapy (TMZ/TMZ+BEV/Others (No TMZ)/No or NA)**	0.883 (0.852-0.913)	**1.06e-04**	0.918 (0.879-0.958)	**2.87e-02**	0.573 (0.432-0.759)	**1.04e-04**	0.663 (0.487-0.901)	**8.80e-03**
**Radiotherapy (No/Yes/NA)**	0.433 (0.262-0.714)	**1.04e-03**	0.273 (0.156-0.476)	**4.69e-06**	0.668 (0.492-0.908)	**9.96e-03**	0.722 (0.682-0.762)	**4.11e-02**
**Surgery (Biopsy only/Tumor resection)**	0.934 (0.523-1.667)	0.82	-	-	NA		NA	
**IDH mutation status (Wild-type/Mutant)**	0.262 (0.096-0.715)	**8.91e-03**	0.279 (0.240-0.318)	**3.24e-02**	0.752 (0.566-0.988)	**3.89e-02**	0.807 (0.767-0.847)	**5.68e-03**
**MGMT promoter status (Methylated/Unmethylated)**	1.434 (1.133-1.733)	**6.84e-03**	1.365 (1.325-1.404)	**1.31e-02**	NA		NA	
**TERT promoter status (Wild-type/Mutant)**	0.906 (0.287-2.861)	0.87	-	-	NA		NA	
**BRAF status (Wild-type/Mutant)**	1.973 (0.720-5.410)	0.19	-	-	NA		NA	
**ATRX status (Wild-type/Mutant)**	0.426 (0.187-0.973)	**4.28e-02**	0.899 (0.703-2.095)	0.32	NA		NA	
**EGFR status (Wild-type/Mutant)**	1.273 (0.873-1.857)	0.21	-	-	NA		NA	
**G-CIMP status (Non- or G-CIMP)**	0.241 (0.088-0.655)	**5.32e-03**	1.474 (0.912-2.043)	0.29				
**Molecular subtype (Classical/Neural/Mesenchymal/Proneural)**	0.971 (0.831-1.133)	0.71	-	-				
**1p/19q status (Noncodeletion/Codeletion/NA)**	NA		NA		0.913 (0.662-1.259)	0.58	-	-
**GDRG signature**	1.332 (1.153-1.540)	**1.05e-04**	1.297 (1.109-1.517)	**1.17e-03**	2.558 (1.815-3.606)	**8.07e-08**	2.609 (1.744-3.902)	**3.03e-06**

GDRG, GBM cell differentiation-related gene; GBM, glioblastoma; NA, not available; HR, hazard ratio; CI, confidence interval; KPS, Karnofsky performance status; TMZ, temozolomide; BEV, bevacizumab; PCV, procarbazine lomustine vincristine.

“Others (no TMZ)” in pharmacotherapy included PCV, PCV+BEV, and other drugs, including avastin, carmustine, and irinotecan.

All statistical tests were two-sided. Bold type indicates P<0.05.

## DISCUSSION

Cancer cells are derived from CSCs, initiate tumor mass growth, and drive tumor progression and invasion forward. They differentiate into diverse subpopulations during differentiation due to hyperproliferation and increased genetic instability [20]. As a result, one group of cells can express heterogeneous phenotypes within a tumor and stay in distinct differentiation states [19, 21]. GBM, the most common and lethal CNS neoplasm, has a highly heterogeneous intratumoral cell composition [22]. Since intratumoral heterogeneity is increasingly considered to be one of the main causes of treatment resistance, there is an urgent need to harness new technologies, including single-cell analysis, to explore cell heterogeneity in GBMs [23]. Several studies have researched the differentiation of GBM CSCs [24–26]. However, to date, studies on the differentiation states of GBM cancer cells are limited. Whether GBM cell differentiation states are correlated with clinical outcomes and the treatment response remains unknown. In this study, we demonstrated that GBM cells could be divided into two subsets with distinct differentiation characteristics, and the classification of patients based on GBM cell differentiation patterns was correlated with patient OS after treatment and the tumor response to immunotherapy. These findings were initially obtained based on scRNA-seq data and then validated using the bulk RNA-seq data of GBM patients from two large databases.

Intratumoral heterogeneity refers to the different features of cells in a single tumor, and these cells manifest as a diverse collection of cells with distinct molecular signatures or differentiation states [27]. In this study, we identified 13 cell clusters in GBMs. Four of them were GBM cells, and 1 cluster was GBM CSCs. One cluster was annotated as astrocytes, one was annotated as oligodendrocytes, and the other 6 clusters were annotated as immune cells, mostly macrophages, which is consistent with the previous literature [28]. According to the trajectory analysis, GBM cells were projected into two subsets with remarkably distinct differentiation features, and subset-dependent GDRGs were identified. Using GSEA and correlation analysis, we found that this differentiation model was associated with immune regulation and metabolic pathways in GBMs, implying that intrinsic correlations between GBM cell differentiation and intratumoral immune and metabolic biology exist.

*EGFR*, observed in approximately 57% of GBMs, acts as a major driver of tumor invasion, progression, and angiogenesis [29–31]. As shown in this study, *EGFR*, identified as a type II GDRG, was the top mutated GDRG, with a mutation frequency of 53%. This finding unveils the role of *EGFR* in regulating GBM cell differentiation and its subsequent role in interacting with tumor immunity and cell fate.

One hallmark of uncontrolled cancer progression and invasion is immune escape [20]. Immunotherapy can relieve the intratumoral immunosuppression status and acts as a promising treatment strategy for GBM patients; it has been previously shown to remarkably improve the survival of patients with several other tumors [5]. Unfortunately, the outcomes of almost all trials for GBMs, including those on immune checkpoint inhibitors, vaccinations, and adoptive T cell therapy, have not been as effective as expected. The low immunogenicity of GBM, the immune privilege of the CNS and the immunosuppressive microenvironment are considered key pathophysiologies underlying the immunotherapy resistance of GBMs [32]. We revealed in this study that type I GDRGs are correlated with intratumoral immunosuppression, disturbing the processes of antigen processing and presentation and T cell differentiation in GBMs. TIDE analysis showed that patients with MC1 GBMs, namely, those harboring more type I GDRGs, were less likely to respond to immunotherapy than patients with MC2 GBMs. The expression of immunotherapy-targeted molecules differed in GBMs with different cell differentiation patterns. Our results showed that MC1 GBMs expressed more PD1/PDL1/PDL2 molecules, while MC2 GBMs expressed more CTLA4/CD80/CD86 molecules. Moreover, the SubMap analysis confirmed that the immunoreaction was related to cell differentiation, predicting that patients with MC1 GBMs were more sensitive to anti-PD1 therapies, while patients with MC2 GBMs were more sensitive to anti-CTLA4 therapies. Therefore, based on these findings, we propose that the features of GBM cell differentiation states can be referred to as a good predictor for the GBM immunotherapy response.

We explored and validated the prognostic predictive value of the GDRGs and their correlations with patient clinical outcomes. The machine learning-based unsupervised clustering method was used in this study to classify GBM patients into two groups based on the cell differentiation states, namely, the GDRG features, of GBM cells. Two large GBM databases of different origins, the TCGA and CGGA, were used, and the survival analysis showed that patients with MC1 GBMs had poorer OS than patients with MC2 GBMs, indicating that the GDRG-based patient classification can be used to predict patient survival.

A nomogram is a multivariable regression model that is widely used in studies to predict clinical outcomes with intuitive visual presentations [33]. In this study, *FN1*, *APOE*, *RPL7A* and *GSTM2* were identified as the 4 most significant survival-predicting GDRGs in human GBMs. We successfully established a risk score formula based on these GDRGs and generated a clinically applicable nomogram with GDRG signatures and clinicopathological parameters to predict GBM patient outcome. We then validated this nomogram in two large GBM cohorts with long-term follow-up examinations, showing the high reliability of this nomogram. To our knowledge, this nomogram is the first to incorporate a cell differentiation-related signature for predicting GBM patient survival. This visualized scoring system may assist neurosurgeons and oncologists in performing survival predictions according to clinicopathological and cell differentiation information and in further proposing better treatment options.

The current study has some limitations. We conducted this analysis and made conclusions using data from published databases, and the prediction model was validated using TCGA and CGGA cohorts but not our own cohort. Additionally, detailed patient information was incomplete, and some clinical parameters, e.g., tumor imaging results, medical records and history, and operation note details, were not available for download and thus were not input into the nomogram. The predictive model needs to be further validated in prospective large-scale cohorts.

## CONCLUSIONS

We used scRNA-seq and bulk RNA-seq data and found that GBM cells follow a two-dimensional differentiation trajectory and that their differentiation states correlate with several immune regulation and metabolic pathways. The classification of GBM patients based on GBM cell differentiation patterns can reliably predict patient survival, immune checkpoint expression, and the tumor immunotherapy response. We identified the key prognosis-predicting GDRGs and established a nomogram composed of patient clinicopathological variables and these GDRGs to predict GBM prognosis. This study highlights the distinct cell differentiation trajectories of GBM cells and their essential roles in predicting the clinical outcome of GBM patients and the tumor immunotherapy response.

## MATERIALS AND METHODS

### Data acquisition and preprocessing

The scRNA-seq data and bulk RNA-seq data of human GBM samples were included in this study for analysis. The scRNA-seq data of a total of 3,589 cells of 4 human primary GBM samples, accession number GSE84465 [34], were obtained from the Gene Expression Omnibus (GEO, http://www.ncbi.nlm.nih.gov/geo/) database, containing 2,343 cells from tumor cores and 1,246 cells from peripheral regions, with a reading depth of 10× genomics based on Illumina NextSeq 500. The bulk RNA-seq profiles of GBM samples were obtained from the TCGA database (https://portal.gdc.cancer.gov/) and the CGGA database (http://www.cgga.org.cn). We excluded samples with unavailable clinical information and ultimately included 151 GBMs from the TCGA cohort as the training set and 350 GBMs from the CGGA cohort as the validation set.

### Processing of the GBM scRNA-seq data

In total, 2,343 cells from tumor cores were included in this analysis. The Seurat package in R 3.5.1 was used for quality control, statistical analysis, and exploration of the scRNA-seq data [35]. First, 194 low-quality cells were excluded based on the following quality control standards: 1) genes detected in < 3 cells were excluded; 2) cells with < 50 total detected genes were excluded; and 3) cells with ≥ 5% of mitochondria-expressed genes were excluded. Then, the gene expression of the remaining 2,149 cells was normalized using a linear regression model. PCA was performed to identify significantly available dimensions with a P value < 0.05 [36]. Then, the t-distributed stochastic neighbor embedding (tSNE) algorithm was applied for dimensionality reduction with 20 initial PCs and for performing cluster classification analysis across all cells [37]. The differential expression analysis among all genes within cell clusters was performed using the limma package in R to identify the marker genes of each cluster. An adjusted P value < 0.05 and | log_2_[fold change (FC)] | > 0.5 were considered the cutoff criteria for identifying marker genes. Afterwards, different cell clusters were determined and annotated by the singleR package according to the composition patterns of the marker genes and were then manually verified and corrected with the CellMarker database [38, 39]. The corresponding genes of cell surface markers for the annotation of cell clusters are listed in Supplementary Table 2.

### Trajectory analysis and GDRG identification

Single-cell pseudotime trajectories of the GBM scRNA-seq data were constructed using the Monocle 2 algorithm [14]. This algorithm adopts a machine learning technique, learning a parsimonious principal graph to reduce the given high-dimensional expression profiles to a low-dimensional space. Single cells were projected onto this space and ordered into a trajectory with branch points. For data interpretation, the cells in the same branch were generally considered to be in the same differentiation state, while cells located in different branches were considered to have different cell differentiation characteristics. In addition, differential expression analysis was performed between branches, and genes that showed differential expression levels were defined as branch-dependent or state-specific genes or marker genes. These marker genes of GBM cells located in different branches were defined as GDRGs.

### GSEA, correlation analysis, and somatic mutation analysis of branch-dependent GDRGs

GSEA (http://software.broadinstitute.org/gsea/index.jsp) was performed to identify the related molecular mechanisms and pathways of GBM cells in different differentiation states [40]. The adjusted P value was used to correct the false positive results by using the default Benjamini-Hochberg false discovery rate (FDR) method. An FDR value ≤ 0.05 of the enrichment gene sets was considered statistically significant. Then, we determined whether the observed cell subtypes generated from the scRNA-seq GBM data could also be identified in the bulk RNA-seq data. Because the coregulation of transcriptional programs in bulk RNA-seq data would indicate a common cellular origin, we believed that cell populations would be best identified by distinguishing gene expression profiles consisting of highly correlated genes [15]. Thus, correlation analyses of the expression of GDRGs were performed using Pearson’s correlation test, and the results were visualized as a heatmap. A P value < 0.05 and |correlation coefficient| > 0.3 were considered significantly correlative. To determine whether the gene profiles originating from different cell populations were functionally correlated, we utilized metagenes to represent the overall expression patterns of the corresponding gene profiles. The relative expression values of metagenes, an assembly of multiple genes, were derived as the weighted averages of the expression levels of the constituent genes using a logistic regression model [15]. Correlation analysis between branch-dependent GBM differentiation-related metagenes was performed by Pearson’s correlation test in both the scRNA-seq data and bulk RNA-seq data. Moreover, we performed somatic mutation analysis to identify the mutation statuses of the GDRGs. The cBioPortal database (https://www.cbioportal.org/) was used to download the somatic mutation data of GBM patients. The numbers of variant types and classifications were visualized with OncoPlot.

### GDRG-based classifications of GBM patients in the TCGA and CGGA cohorts

Unsupervised consensus clustering, an algorithm based on k-means machine learning, was utilized to explore a molecular classification of both the TCGA and CGGA GBM cohorts based on the expression patterns of GDRGs using the ‘ConsensusClusterPlus’ package in R [41]. The clustering procedure, with 1,000 iterations, was performed by sampling 80% of the data in each iteration. The optimal number of clusters was determined by the relative change in the area under the CDF curves of the consensus score and consensus heatmap. Then, Kaplan-Meier survival analysis was performed to evaluate the prognosis of patients in different MCs. We also performed comparisons of the clinicopathological variables between different clusters of patients to further explore the associations between the GDRG-based MCs and the clinical features of GBM patients.

### Immunotherapy response predictions

TIDE (http://tide.dfci.harvard.edu/) is a computational method that integrates the expression signatures of T cell dysfunction and exclusion to model tumor immune evasion [42]. We used the TIDE algorithm to predict the clinical response to immune checkpoint blockade (ICB) in GBM patients based on pretreatment genomics. The unsupervised subclass mapping method (SubMap; https://cloud.genepattern.org/gp/) was further applied to predict the response of GBM patients in different MCs to immunotherapy, including anti-PD1 and anti-CTLA4 therapy [43]. A Bonferroni-corrected P value < 0.05 was considered statistically significant.

### Generation and validation of the GDRG-based prognostic risk score model

The associations between the expression levels of GDRGs and patient survival were first evaluated by univariate Cox regression analysis in the TCGA training cohort. The prognosis-related genes with a P value < 0.05 identified by the analysis were further screened by LASSO and multivariate Cox regression analyses. Consequently, a risk score model based on the key prognosis-related GDRGs was constructed to predict the prognosis of GBM patients [44]. We calculated the risk score of each patient by referring to our previously constructed formula, Risk score = Exp_*GENE1*_ × β1 + Exp_*GENE2*_ × β2 +…+ Exp_*GENEn*_ × βn, in which “Exp” represents the expression level of the corresponding gene, and “β” represents the regression coefficient calculated by the multivariate Cox analysis [9]. Accordingly, all GBM patients in the TCGA database were stratified into either the low-risk (low score) group or the high-risk (high score) group. Kaplan-Meier survival analysis was performed to estimate the OS of these two groups, and survival differences were evaluated by a two-sided log-rank test. The predictive accuracy of the GDRG-based prognostic model was evaluated by Harrell's concordance index (C-index) and time-dependent ROC curve analysis within 0.5, 1 and 3 years by utilizing the survcomp and survivalROC packages in R [33, 45]. Both the C-index and AUC ranged from 0.5 to 1, with 1 indicating perfect discrimination and 0.5 indicating no discrimination. Finally, the prognostic model generated by the TCGA training cohort was verified in the CGGA validation cohort.

Afterwards, univariate and multivariate Cox regression analyses were performed in both GBM cohorts to determine whether the predictive performance of the GDRG signatures could be independent of the clinicopathological variables. These variables included age, sex, Karnofsky performance status score, pharmacotherapy, radiotherapy, surgery, isocitrate dehydrogenase (IDH) mutation status, O6-methylguanine-DNA-methyltransferase (MGMT) promoter methylation status, telomerase reverse transcriptase (TERT) promoter mutation status, B-Raf proto-oncogene (BRAF) mutation status, X-linked alpha thalassemia mental retardation syndrome gene (ATRX) mutation status, EGFR mutation status, glioma CpG island methylator phenotype (G-CIMP) status, molecular subtype (classical, neural, mesenchymal, and proneural), and 1p/19q status.

### Development and validation of the prognostic nomogram with the GDRG signature and clinicopathological parameters

Following univariate and multivariate Cox regression analyses, all the identified independent prognostic parameters were utilized to develop a prognostic nomogram for predicting the 0.5-, 1-, and 3-year survival outcomes of GBM patients using the rms package in R. Calibration plots at 0.5, 1, and 3 years were constructed to graphically evaluate the discriminative ability of the nomogram [45]. Then, the discrimination performance of the nomogram was quantitatively assessed by the C-index and ROC curve [33]. Finally, the nomogram constructed using the data from the TCGA cohort was validated in the CGGA cohort.

### Data accessibility

The scRNA-seq data of GBM samples (accession number GSE84465) were obtained from the GEO database (http://www.ncbi.nlm.nih.gov/geo/). The bulk RNA-seq profiles of GBM samples were obtained from the TCGA database (https://portal.gdc.cancer.gov/) and the CGGA database (http://www.cgga.org.cn).

### Ethics approval

Ethics committee approval for our study was not required because the data were obtained from publicly available databases.

## Supplementary Material

Supplementary Figures

Supplementary Tables
