# Supplementary material for: Glioblastoma cell differentiation trajectory predicts the immunotherapy response and overall survival of patients
Source: Aging (Albany NY). 2020 Sep 21;12(18):18297–321. doi: 10.18632/aging.103695 (PMC7585071; doi:10.18632/aging.103695)
Supplement: Supplementary Figures [file aging-12-103695-s002..pdf]

SUPPLEMENTARY FIGURES

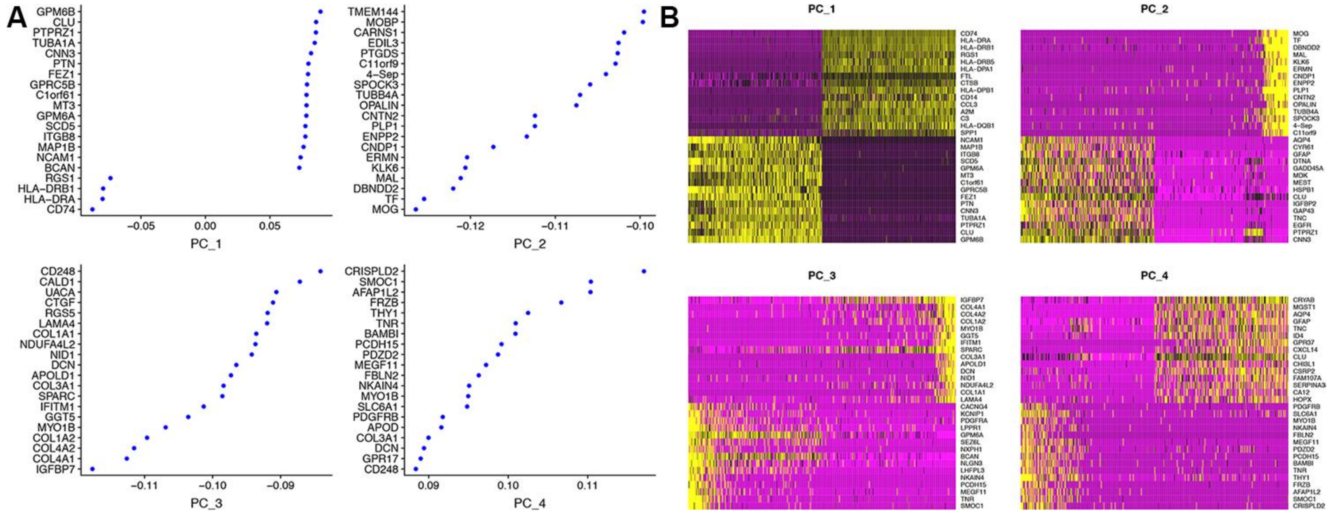

Supplementary Figure 1. The top 20 significantly correlated genes in PC_1 to PC_4 are displayed using dot plots and heatmaps. (**A**) The dot plots show the top 20 significantly correlated genes in each component. The X axis represents the correlation coefficient. (**B**) The heatmaps show the expression patterns of the top 20 significantly correlated genes in each component. The colors from purple to yellow indicate the gene expression levels from low to high.

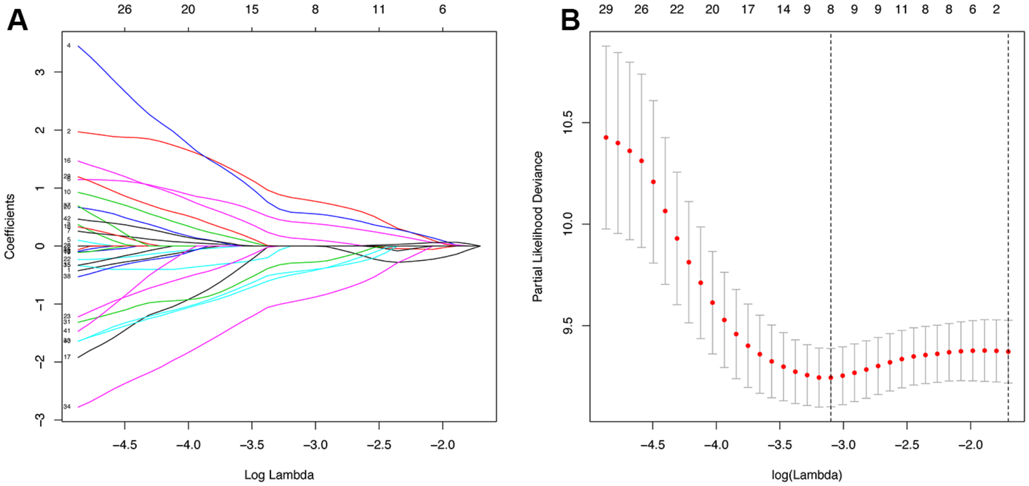

C

Gene	Coefficient	HR	95%CI	Pvalue
**FN1**	1.664706394	5.284121573	2.240943053-12.45990645	0.000142608
**APOE**	-0.930625639	0.394306939	0.138469339-1.122833136	0.001337481
**RPL7A**	-1.296966955	0.273359649	0.091137625-0.819919298	0.020654232
**GSTM2**	-0.896694361	0.407915856	0.172035503-0.967215154	0.041786704

Supplementary Figure 2. The prognosis-related GDRGs were screened with LASSO and multivariate Cox regression analyses. (**A**) The coefficient profile plot was produced against the log(lambda) sequence. A vertical line was drawn at the value selected using ten-fold cross-validation, where an optimal lambda value resulted in ten features with nonzero coefficients. (**B**) Optimal parameter (lambda) selection in the LASSO model used ten-fold cross-validation via a minimum criteria. The partial likelihood deviance (binomial deviance) curve was plotted versus the log(lambda) value. Dotted vertical lines were drawn at the optimal values by using the minimum criteria and the l standard error of the minimum criteria. (**C**) Following LASSO regression analysis, 4 GDRGs were identified with multivariate Cox regression analysis.

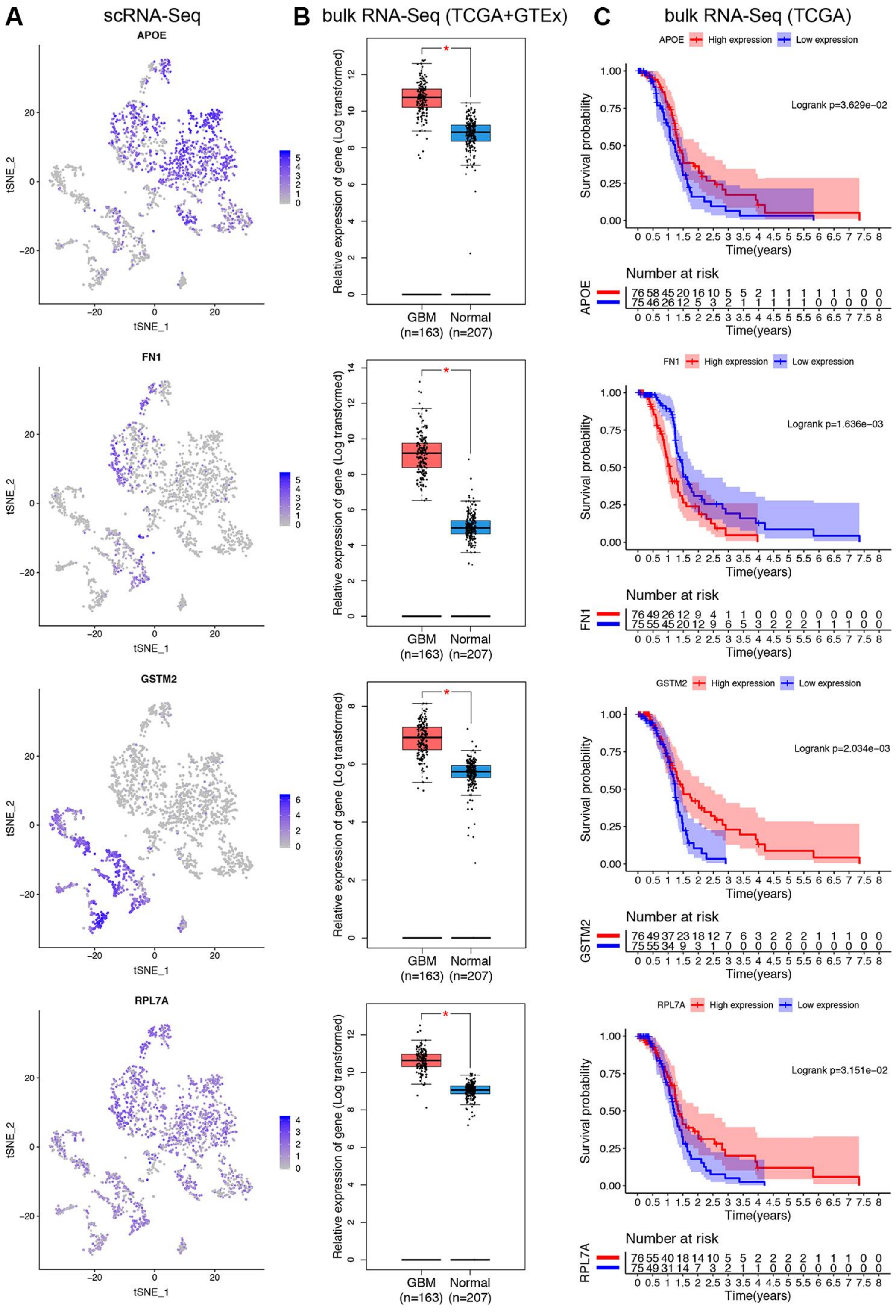

Supplementary Figure 3. Expression and survival analyses of the four GDRGs in the prediction model. (**A**) The expression of the four GDRGs in scRNA-seq data is shown. The colors from gray to blue indicate the gene expression levels from low to high. (**B**) The expression of the four GDRGs in bulk RNA-seq data, including 163 GBM (TCGA) and 207 normal (GTEx) samples, is shown. Asterisks indicate $p < 0.05$. (**C**) K-M survival analyses demonstrated that the expression of GDRGs was associated with OS in TCGA GBM patients.
