# Supplementary material for: Glioblastoma cell differentiation trajectory predicts the immunotherapy response and overall survival of patients
Source: Aging (Albany NY). 2020 Sep 21;12(18):18297–321. doi: 10.18632/aging.103695 (PMC7585071; doi:10.18632/aging.103695)
Supplement: Supplementary Tables [file aging-12-103695-s001..pdf]

SUPPLEMENTARY TABLES

Supplementary Table 1. The marker genes of type I and type II GBM cell subsets.

GBM cell subsets	GBM cell differentiation-related genes (GDRGs)
**Type I GDRGs (n=265)**	A2M, ABL2, ACSL1, ADAM28, ADAP2, ADORA3, AIF1, AKAP13, ALOX5, ALOX5AP, ANXA1, APBB1IP, APOC1, APOE, ARAP1, ARHGAP18, ARHGAP4, ARHGDIB, ARNT2, ARPC1B, ARPC2, ATP6V0E2, B3GNT5, B4GALT1, BAALC, BCL2A1, C10orf54, C1orf162, C1orf38, C1QA, C1QB, C1QC, C3, C3AR1, C5AR1, CAPG, CCL3, CCL4, CD14, CD163, CD300A, CD4, CD53, CD68, CD74, CD83, CD84, CD86, CD93, CDKN1A, CHL1, CLEC7A, CORO1A, CPE, CSF1R, CSF3R, CTSB, CTSC, CTSH, CTSL1, CTSS, CXCR4, CYBA, CYBB, CYTH4, DAG1, DDR1, DENND3, DKK3, DOCK2, DOCK8, DSE, ELL2, ENG, ETS2, ETV1, EVI2B, F13A1, FCER1G, FCGBP, FCGR1A, FCGR2A, FCGR3A, FCGRT, FERMT3, FGD4, FGL2, FHL1, FPR1, FTH1, FTL, FXYD5, FYB, FYN, GATM, GK, GMFG, GNA13, GOLM1, GPM6B, GPNMB, GPR183, GPRC5B, GPX1, HAMP, HAVCR2, HCK, HCLS1, HLA-B, HLA-DMA, HLA-DMB, HLA-DPA1, HLA-DPB1, HLA-DQA1, HLA-DQB1, HLA-DRA, HLA-DRB1, HLA-DRB5, HMHA1, IER3, IFI30, IFNGR1, IL10RA, IL13RA1, IL18, IL8, INPP5D, IRF8, ITGAM, ITGAX, ITGB2, ITGB8, ITM2C, ITPRIP, KCTD12, KLF6, LAIR1, LAPTM5, LAT2, LCP1, LCP2, LGALS9, LILRB4, LIMS1, LPCAT2, LST1, LY86, LYN, LYZ, MAGED1, MANBA, MAP3K8, MEF2C, MERTK, METRNL, MGAT1, MPP1, MS4A4A, MS4A6A, MS4A7, MSR1, MYO1F, MYO9B, NAV2, NCF4, NFKBID, NINJ1, NPC2, NR4A2, NR4A3, NRCAM, NTRK2, OLR1, OTUD1, PABPC1, PABPC4, PARVG, PFN1, PIK3AP1, PIK3R5, PLAUR, PLCB2, PLEK, PLIN2, PLXDC2, PMP2, PTAFR, PTGER4, PTPN6, PTPRC, PTPRZ1, RANBP2, RAPGEF1, REL, RGS1, RGS10, RHBDF2, RHOG, RILPL2, RIN3, RNASE6, RNASET2, RNF144B, RNF149, RPL23, RPS23, RPS24, RPS3, RPS6, S100A11, S100A4, SAMSN1, SAT1, SCIN, SCRG1, SDC3, SELPLG, SERPINA1, SERPINB9, SERPINE2, SH3BGRL3, SH3TC1, SIGLEC10, SLA, SLC11A1, SLC1A2, SLC22A17, SLC2A5, SLC31A2, SLCO2B1, SOD2, SORBS1, SPARCL1, SPP1, SRGN, STAB1, STAT6, SYK, SYNGR2, SYT11, TBXAS1, TCIRG1, TFRC, TGFBI, THBS2, TLR2, TNFRSF1B, TPM3, TPT1, TREM1, TREM2, TRIM9, TRO, TSPAN3, TSPAN7, TYMP, TYROBP, UCHL1, UCP2, UPP1, USP36, VAMP8, VSIG4, ZBTB20, ZNF331
**Type II GDRGs (n=193)**	ANPEP, RNASE1, ADAM8, GPNMB, MARCO, FTL, FCGR2B, C15orf48, LSP1, EGR1, TGFBI, S100A10, S100A4, LYZ, TMSB10, LGALS1, S100A9, CD109, SLC16A10, FTH1, ANXA2, VIM, TPT1, CSTB, IFI30, CYTIP, FBP1, BCAT1, GAPDH, SH3BGRL3, LCP1, GPR56, LDHA, RPS18, NDRG1, FLNB, TGM2, ERO1L, PLIN2, VCAN, TNFRSF14, SLC1A3, PAPSS2, RPS24, ENO1, NCF2, TCIRG1, BTG1, TXNIP, LGALS3, AHNAK, CTSD, S100A6, UPP1, ALOX5AP, BRI3, HK2, TYROBP, CLEC5A, TREM1, CD163, RPS6, DBI, FN1, PKM2, FOS, ANXA1, LAPTM5, CD44, S100A11, RPS2, RPL37, TUBGCP2, RPS9, TAGLN2, YBX1, METRNL, RPL37A, RPS3, TRIM25, RPL27A, ALDOA, RPL31, C1orf61, CNN3, ST14, CXCL2, PTN, CLU, CD68, RPL23, CKB, RPS4Y1, TYMP, PTK2B, EEF1A1, MGAT1, RPL9, RPL13, JARID2, S100A8, NFIA, MAP1B, ARHGAP18, RPS12, MAPK13, RPL32, MMP19, NCF1, ITGAM, RPL12, CAPG, PTPRZ1, PON2, GPM6B, UBA52, SPP1, BNIP3, TBC1D2, TJP1, EIF4EBP1, NFIB, PGK1, OAZ1, GFAP, GSTO1, RPLP0, ATP1B2, EEF1G, RPS23, ID2, GPM6A, G0S2, CALM2, HMOX1, FNDC3B, TUBB2B, EGFR, DMXL2, GSTM2, BNIP3L, CALD1, TNS1, RPS8, CTSB, H2AFY, C3, LRRFIP1, BTG2, PMP2, FEZ1, ITGB8, CHL1, ARPC2, P4HB, NRCAM, GPI, SLC39A8, RIPK2, FGR, ANGPTL4, AASS, CTSL1, ITM2C, ITGAX, SLC11A1, RPSA, SRI, RPL8, SCD5, GPRC5B, EGR3, RPL7A, SLCO4A1, RPL13A, GRB2, SCRG1, CX3CR1, GLUL, BCAN, ZBTB20, DDX5, DTNA, DHRS3, SDS, MEST, F3, IL4I1, MT3, FHL1, DDR1, TCF4, COTL1, FXYD6, PFN2, ABCA1, XIST, TUBA1A, CBX5, IER2, MAGED1, RAMP1, PIM1, SDC2, GSTM3, HTRA1, CRYAB, SLC2A1, MARCKSL1, SPRY2, MDK, KIAA1274, CHD7, GATM, CH25H, OLFML3, DDX41, TSPAN3, LONP1, PADI2, SPTBN1, TMEM9, HSPA6, NDUFA4, RCN2, NDRG2, WRB, ZFP36, LIMA1, STMN1, DPYSL2, SBDS, NGFRAP1, FAM46A, JUNB, SPARC, SKP1, SERPING1, FOSB, SRGAP1, SYT11, GADD45A, SDC3, GABARAPL2, AHCYL1, NUPR1, METTL9, DUSP1, EGR2, ITPR2, CBR1, PGRMC1, S100B, CCND2, TUBA1B, TSPAN31, PRDX2, TSC22D4, LPL, MLF2, TMX2, 7-Sep, RBBP4, PEBP1, RHBDD2, EIF4E, NR4A3, SPARCL1, ST6GAL1, RTN3, CCL4, EID1, CASC4, ATP5A1, TSC22D1, LDHB, SOD1, CCT6A, CCL2, CDK4, KPNA2, RASSF4, CLDND1, A2M, SORL1, LGALS3BP, CNP, CREB5, GBAS, HSPB1, IL1B, CLIC4, NR4A1
**Total GDRGs (n=498)**	ANPEP, RNASE1, ADAM8, GPNMB, MARCO, FTL, FCGR2B, C15orf48, LSP1, EGR1, TGFBI, S100A10, S100A4, LYZ, TMSB10, LGALS1, S100A9, CD109, SLC16A10, FTH1, ANXA2, VIM, TPT1, CSTB, IFI30, CYTIP, FBP1, BCAT1, GAPDH, SH3BGRL3, LCP1, GPR56, LDHA, RPS18, NDRG1, FLNB, TGM2, ERO1L, PLIN2, VCAN, TNFRSF14, SLC1A3, PAPSS2, RPS24, ENO1, NCF2, TCIRG1, BTG1, TXNIP, LGALS3, AHNAK, CTSD, S100A6, UPP1, ALOX5AP, BRI3, HK2, TYROBP, CLEC5A, TREM1, CD163, RPS6, DBI, FN1, PKM2, FOS, ANXA1, LAPTM5, CD44, S100A11, RPS2, RPL37, TUBGCP2, RPS9, TAGLN2, YBX1, METRNL, RPL37A, RPS3, TRIM25, RPL27A, ALDOA, RPL31, C1orf61, CNN3, ST14, CXCL2, PTN, CLU, CD68, RPL23, CKB, RPS4Y1, TYMP, PTK2B, EEF1A1, MGAT1, RPL9, RPL13, JARID2, S100A8, NFIA, MAP1B, ARHGAP18, RPS12, MAPK13, RPL32, MMP19, NCF1, ITGAM, RPL12, CAPG, PTPRZ1, PON2, GPM6B, UBA52, SPP1, BNIP3, TBC1D2, TJP1, EIF4EBP1, NFIB, PGK1, OAZ1, GFAP, GSTO1, RPLP0, ATP1B2, EEF1G, RPS23, ID2, GPM6A, G0S2, CALM2, HMOX1, FNDC3B, TUBB2B, EGFR, DMXL2, GSTM2, BNIP3L, CALD1, TNS1, RPS8, CTSB, H2AFY, C3, LRRFIP1, BTG2, PMP2, FEZ1, ITGB8, CHL1, ARPC2, P4HB, NRCAM, GPI, SLC39A8, RIPK2, FGR, ANGPTL4, AASS, CTSL1, ITM2C, ITGAX, SLC11A1, RPSA, SRI, RPL8, SCD5, GPRC5B, EGR3, RPL7A, SLCO4A1, RPL13A, GRB2, SCRG1, CX3CR1, GLUL, BCAN, ZBTB20, DDX5, DTNA, DHRS3, SDS, MEST, F3, IL4I1, MT3, FHL1,

DDR1, TCF4, COTL1, FXYD6, PFN2, ABCA1, XIST, TUBA1A, CBX5, IER2, MAGED1, RAMP1, PIM1, SDC2, GSTM3, HTRA1, CRYAB, SLC2A1, MARCKSL1, SPRY2, MDK, KIAA1274, CHD7, GATM, CH25H, OLFML3, DDX41, TSPAN3, LONP1, PADI2, SPTBN1, TMEM9, HSPA6, NDUFA4, RCN2, NDRG2, WRB, ZFP36, LIMA1, STMN1, DPYSL2, SBDS, NGFRAP1, FAM46A, JUNB, SPARC, SKP1, SERPING1, FOSB, SRGAP1, SYT11, GADD45A, SDC3, GABARAPL2, AHCYL1, NUPR1, METTL9, DUSP1, EGR2, ITPR2, CBR1, PGRMC1, S100B, CCND2, TUBA1B, TSPAN31, PRDX2, TSC22D4, LPL, MLF2, TMX2, 7-Sep, RBBP4, PEBP1, RHBDD2, EIF4E, NR4A3, SPARCL1, ST6GAL1, RTN3, CCL4, EID1, CASC4, ATP5A1, TSC22D1, LDHB, SOD1, CCT6A, CCL2, CDK4, KPNA2, RASSF4, CLDND1, A2M, SORL1, LGALS3BP, CNP, CREB5, GBAS, HSPB1, IL1B, CLIC4, NR4A1, ABL2, ACSL1, ADAM28, ADAP2, ADORA3, AIF1, AKAP13, ALOX5, APBB1IP, APOC1, APOE, ARAP1, ARHGAP4, ARHGDIB, ARNT2, ARPC1B, ATP6V0E2, B3GNT5, B4GALT1, BAALC, BCL2A1, C10orf54, C1orf162, C1orf38, C1QA, C1QB, C1QC, C3AR1, C5AR1, CCL3, CD14, CD300A, CD4, CD53, CD74, CD83, CD84, CD86, CD93, CDKN1A, CLEC7A, CORO1A, CPE, CSF1R, CSF3R, CTSC, CTSH, CTSS, CXCR4, CYBA, CYBB, CYTH4, DAG1, DENND3, DKK3, DOCK2, DOCK8, DSE, ELL2, ENG, ETS2, ETV1, EVI2B, F13A1, FCER1G, FCGBP, FCGR1A, FCGR2A, FCGR3A, FCGRT, FERMT3, FGD4, FGL2, FPR1, FXYD5, FYB, FYN, GK, GMFG, GNA13, GOLM1, GPR183, GPX1, HAMP, HAVCR2, HCK, HCLS1, HLA-B, HLA-DMA, HLA-DMB, HLA-DPA1, HLA-DPB1, HLA-DQA1, HLA-DQB1, HLA-DRA, HLA-DRB1, HLA-DRB5, HMHA1, IER3, IFNGR1, IL10RA, IL13RA1, IL18, IL8, INPP5D, IRF8, ITGB2, ITPRIP, KCTD12, KLF6, LAIR1, LAT2, LCP2, LGALS9, LILRB4, LIMS1, LPCAT2, LST1, LY86, LYN, MANBA, MAP3K8, MEF2C, MERTK, MPP1, MS4A4A, MS4A6A, MS4A7, MSR1, MYO1F, MYO9B, NAV2, NCF4, NFKBID, NINJ1, NPC2, NR4A2, NTRK2, OLR1, OTUD1, PABPC1, PABPC4, PARVG, PFN1, PIK3AP1, PIK3R5, PLAUR, PLCB2, PLEK, PLXDC2, PTAFR, PTGER4, PTPN6, PTPRC, RANBP2, RAPGEF1, REL, RGS1, RGS10, RHBDF2, RHOG, RILPL2, RIN3, RNASE6, RNASET2, RNF144B, RNF149, SAMSN1, SAT1, SCIN, SELPLG, SERPINA1, SERPINB9, SERPINE2, SH3TC1, SIGLEC10, SLA, SLC1A2, SLC22A17, SLC2A5, SLC31A2, SLCO2B1, SOD2, SORBS1, SRGN, STAB1, STAT6, SYK, SYNGR2, TBXAS1, TFRC, THBS2, TLR2, TNFRSF1B, TPM3, TREM2, TRIM9, TRO, TSPAN7, UCHL1, UCP2, USP36, VAMP8, VSIG4, ZNF331

Supplementary Table 2. The corresponding genes of cell surface markers (CellMarker database) for identifying cell types of the 13 cell clusters.

Cell populations	Gene symbols of cell surface markers
**Astrocyte**	ALDH1L1, WIF1, NTSR2, GFAP, SOX9, CD40, CD80, CD86, ABCA1, NFIA, S100B, C5AR1, ACSL6, ADCYAP1R1, AGT, ALDOC, ANLN, APOE, ARHGEF26, ATF3, ATP13A4, ATP1B2, BBOX1, BTG2, C1orf61, CA12, CASQ1, CBS, CDC42EP4, CHST9, CLU, CPE, CPNE5, CPVL, CRYAB, CST3, DAND5, DGKG, DKK3, DNJB1, DNASE2, DOK5, DTNA, EDNRB, EEPD1, EFEMP1, EGLN3, EPAS1, EZR, F3, FOS, FOSB, FTH1, FXYD7, GABRB1, GADD45B, CASTOR1, GLUL, GPR75, GRAMD2B, GRIA1, HEPACAM, HEPN1, HEY1, HIF1A, HIF3A, HLA-E, HNMT, HSPB8, ID1, ID4, IER2, IGFBP7, IL11RA, IL33, JUN, JUNB, JUND, KCNH7, KCNIP2, KIF21A, L1CAM, LCAT, LHFPL6, LIX1, LPL, LRIG1, LRRC8A, LYPD1, MAFB, METTL7A, MLC1, MMD2, MT1X, MT2A, MTHFD2, NDRG2, NFKBIA, NHSL1, NANOG, NRP1, NTRK2, P2RY1, PAPLN, PEA15, PER1, PFKFB3, PLTP, PON2, PRLHR, PRRT2, PSAP, RASL10A, RASSF4, RFX4, RGMA, RHOB, RND3, SCG2, SCG3, SEMA6A, SERPINA3, SF3A1, SLC1A2, SLC1A3, SLC39A11, SORL1, SPARCL1, SPOCK1, SPON1, SRPX, SSTR2, ST6GAL2, TACR1, TBC1D10A, TIMP3, TMEM151B, TNS1, TOB2, TPCN1, TRIL, TSC22D4, TSPAN12, ZFAND5, ZFP36, ZFP36L1, ZFP36L2, AQP4, BMPR1B, C16orf89, CHRDL1, CTH, CYBRD1, FGFR3, GLI3, GLIS3, HGF, ITGA7, ITGB4, NWD1, PAQR6, PPP1R3C, RNF43, SLC14A1, SLC30A10, SLC4A4, SORCS2, TRIM66
**Oligodendrocyte**	MBP, OPALIN, RTN4, CSPG4, PDGFRA, OLIG2, ACAT2, ADAMTS4, LHFPL3, ACTG1, AFAP1L2, AMOTL2, ANGPTL2, APOD, ARL4A, ASIC1, ATCAY, ATP5F1E, BIN1, LRRC75A, C2orf27A, CDH13, CDHR1, CNP, COX7C, CRB1, CXADR, DHCR24, DHCR7, DLL1, DLL3, EBP, EEF1B2, EEF2, EIF3E, EIF3L, EPN2, FA2H, FABP7, FAU, FDPS, FERMT1, FGFBP3, FXYD6, GAP43, RACK1, GPR17, GPR37L1, GRIA2, GRIA4, GRIK2, HIP1, HIPK2, IFITM10, KCNIP3, KLRC2, LDHB, LIMA1, LIMS2, LMF1, PLPPR1, LRRN1, MAML2, MAP1A, MAP2, MARCKSL1, MEST, MICAL1, MIF, MTSS1, MYT1, NACA, NAP1L1, NEU4, NME1, NME2, NPM1, NPPA, NXPH1, OLIG1, OMG, OPCML, P2RX7, PGRMC1, PHACTR3, PHLDA1, PHLDB1, PID1, POLR2F, RAB2A, RAB33A, RGMB, RGR, RPL13A, RPL31, RPS17, RPS2, RPS23, RPSAP58, RTKN, SCD, SERINC5, SGK1, SHD, SHISA4, SIRT2, SLC1A1, SNX1, SNX22, SOX8, TAGLN3, TCF12, THY1, TM7SF2, TMEFF2, TMEM97, TMSB10, TMSB4X, TNK2, TRAF4, TUBB, TUBB3, TUBB4A, UGT8, UQCRB, VIPR2, WSCD1, ZCCHC24, ZDHHC9, ZEB2, ASPA, CDK18, CLDN11, DAAM2, DPYD, ERMN, GJB1, GPR37, GRM3, GSN, KCNH8, KLK6, LGI3, LPAR1, MAG, MAL, MAP6D1, MOBP, PLEKHB1, PLP1, PPP1R14A, SEC14L5, SHC4, MEGF11, PCDH15
**Neuron**	L1CAM, DCLK3, RBFOX3, GFAP, MAP2, NES, MARK4, ENO2, SYP, ARMH4, CDO1, CNTN4, COBL, DCN, DLX1, DLX2, DLX5, DLX6, DPYSL5, KDR, GLRA2, GRIA3, KCNK1, KIAA1324, LNX1, LRRTM3, NELL1, NFASC, NXPH1, PNOC, RELN, SLC10A4, SLITRK1, SST, TMEM130
**Glial cell**	GFAP, S100 family
**GBM stem cell (GSC)**	PROM1, CADM1, CLCC1, HMOX1, SCAMP3, SLC16A1, FUT4, CDH5, NANOG, NES, SOX2, THY1, MSI1, CD133, CD15, L1CAM, CD90, A2B5, POU3F2, OLIG2, SALL2
**GBM cell**	PARP1, MBTPS2, CD44
**Endothelial cell**	PECAM1, CDH5, VWF, VCAM1, A2M, APOLD1, FLT1, TM4SF1
**B cell**	MS4A1, CD19, CD79A
**T cell (general)**	CD3D, CD3E, CD3G, CD2
**Regulatory T (Treg) cell**	IL2RA, CD4, FOXP3, CCR8, STAT5B, TGFB1
**T helper-1 (Th1) cell**	CCR6, CXCR3, TBX21, STAT4, STAT1, IFNG, TNF
**T helper-2 (Th2) cell**	GATA3, STAT6, STAT5A, IL13, CCR6, CXCR3, TNFRSF8
**T helper-17 (Th17) cell**	STAT3, IL17A, CCR6, CXCR3
**Tumor-associated macrophage (TAM)**	CD68, CCL2, IL10, AIF1, ARG2, BHLHE40, CD74, CD93, CIB1, CIITA, CREM, CYBB, CYTH1, DOK3, DSE, EMB, FAM49A, FGR, FOSL2, FPR3, FXYD5, GPR132, GPR65, HLA-DMB, HLA-DQA1, HLA-DRB5, IFITM2, IL1RN, IQGAP1, ITGA4, KYNU, LYZ, METRNL, MS4A6A, MS4A7, MXD1, NFIL3, PDE4B, PIM1, PLAC8, PLBD1, PLTP, PQLC3, PTPN7, S100A11, SAMHD1, SH3BGRL, SPINT2, SYNGR2, TGFBI, THBD, TMEM123, TNFSF13, TREM1, VOPP1
**M1 macrophage**	TSPO, IRF5, PTGS2, NOS2
**M2 macrophage**	CD163, VSIG4, MS4A4A
**Monocyte**	CD86, CSF1R
**Neutrophil**	MPO, CEACAM8, ITGAM, CCR7
**Natural killer (NK) cell**	KIR2DL1, KIR2DL3, KIR2DL4, KIR3DL1, KIR3DL2, KIR3DL3, KIR2DS4
**Dendritic cell (DC)**	HLA-DPB1, HLA-DQB1, HLA-DRA, HLA-DPA1, CD1C, NRP1, ITGAX
